# Enhancing LDD diagnosis with YOLOv9-AID: simultaneous detection of pfirrmann grading, disc herniation, HIZ, and Schmorl’s nodules

**DOI:** 10.3389/fbioe.2025.1626299

**Published:** 2025-09-10

**Authors:** Erling Xiang, Yongkang Zou, Jiale Chen, Jian Peng, Chunhai Huang, Feiwen Li, Xiaoping Li, Shenghua Qin, Zhiyu Li, Nanyu Li, Xu Zhou, Mingzheng Zhang

**Affiliations:** ^1^ Department of Radiology, The First Affiliated Hospital of Jishou University, Jishou, China; ^2^ College of Communication Engineering, Jilin University, Changchun, China; ^3^ Department of MRI, The First Affiliated Hospital of Jishou University, Jishou, China; ^4^ Department of Neurosurgery, The First Affiliated Hospital of Jishou University, Jishou, China; ^5^ Department of Orthopaedics, The First Affiliated Hospital of Jishou University, Jishou, China; ^6^ School of Medicine, Jishou University, Jishou, China

**Keywords:** lumbar intervertebral disc degeneration, intelligent diagnosis, MRI images, deep learning, YOLO model

## Abstract

This study develops an intelligent diagnostic model for LDD based on a novel YOLOv9-AID detection network and evaluates the impact of multiple innovative strategies on detection performance. A total of 222 adult patients who underwent lumbar MRI for low back pain or radicular leg pain were enrolled, yielding 1110 de-identified sagittal T2-weighted images (five per case). After excluding cases with prior spinal trauma, tumors, postoperative changes, congenital malformations, or severe artefacts, 202 cases (1,010 images) were randomly divided into training, validation, and internal test sets (8:1:1), while 20 cases (100 images) formed an external dataset for generalization assessment. The YOLOv9-AID model introduces three key enhancements to the baseline YOLOv9: a SlideLoss function to rebalance training weights between high- and low-quality samples; spatial-channel collaborative attention modules (SCSA) embedded at layers 5 and 11 to strengthen lesion feature extraction; and an ExtraDW-inspired redesign of the ResNCSPELAN4 module to boost precision and reduce parameter count. In the internal test set, the model achieved an mAP50 of 82.8% and an overall detection precision of 80.3%, with Schmorl’s node detection at 92.9%, Pfirrmann grading accuracy at 93.3%, and disc herniation accuracy at 73.2% (an 8.4% improvement). Recall rates increased by approximately 5% on average, with Schmorl’s node recall up 15.1%, Pfirrmann recall up 1.8%, and herniation recall improvements of up to 12.3%. External validation confirmed robust generalization, and detection rates for small lesions such as high-intensity zones and Schmorl’s nodes significantly outperformed conventional methods. These results demonstrate that the YOLOv9-AID network, through the integration of SlideLoss and spatial-channel attention mechanisms, substantially enhances the accuracy and robustness of LDD detection on MRI and offers a promising tool to support clinical diagnosis efficiency and consistency.

## 1 Introduction

As a crucial mechanical cushioning structure of the spine, the intervertebral disc plays an essential role, and its degeneration has become one of the leading causes of disability worldwide (Compte et al., 2023). Epidemiological studies indicate that approximately 80% of adults will experience at least one episode of low back pain caused by intervertebral disc pathology during their lifetime (Sood et al., 2023), with lumbar disc degeneration (LDD) and lumbar disc herniation (LDH) being the most common pathological bases (He et al., 2025). These conditions not only significantly affect the quality of life of patients through chronic pain but also lead to loss of work capacity due to restricted mobility, resulting in a substantial socioeconomic burden (Compte et al., 2023; Tsuchiya et al., 2024). The clinical diagnosis is heavily based on magnetic resonance imaging (MRI) (Chu and Sabourdy, 2023); however, traditional manual interpretation faces notable limitations: radiologists must analyze features such as disc height and signal intensity layer by layer on T2-weighted images (T2-WI), with an average evaluation time of 14–19 min per case (Compte et al., 2023; Tsuchiya et al., 2024), and the agreement between observers for the Pfirrmann classification is only at a moderate level (kappa values ranging from 0.66 to 0.89) (Tsuchiya et al., 2024; Soydan et al., 2024). More critically, the global growth rate of radiologists is significantly behind the increasing demand for imaging examinations (Natalia et al., 2024), and training a specialist capable of accurately identifying disc herniation, high intensity zones (HIZ), and Schmorl’s nodes typically requires 5–7 years of clinical experience (Xu et al., 2024). Therefore, developing automated algorithms for LDD detection not only has the potential to reduce diagnostic time to a matter of seconds but also to maintain expert-level reading consistency (classification accuracy > 85%) through deep learning models, which holds significant clinical value for optimizing medical resource allocation and enabling early diagnosis and treatment (Wang et al., 2025).

In the field of LDD diagnosis, deep learning imaging algorithms have indeed widely adopted the You Only Look Once (YOLO) series models for object detection. As a one-stage real-time object detection algorithm, YOLO’s core advantage lies in transforming the object detection task into a regression problem through end-to-end training, thereby achieving a favorable balance between high detection speed and good accuracy (Prisilla et al., 2023). To ensure computational efficiency, YOLO models typically use bounding boxes to annotate target regions. Although this approach simplifies the annotation process, the naive YOLO models present clear limitations. First, existing methods do not fully consider the weight distribution of texture features within the target region. In lumbar MRI images, key pathological features—such as regions relevant to Pfirrmann grading, disc herniation, HIZ, and Schmorl’s nodes—often exhibit localized and distinctive texture characteristics. The traditional rectangular bounding box annotation in YOLO models tends to cause dispersed attention, making it difficult for the model to focus on diagnostically valuable microstructures (Wang et al., 2025) effectively. For example, in LDD classification tasks, the difference in texture between the nucleus pulposus and the annulus fibrosus is crucial for accurate classification, but rectangular classification boxes introduce substantial irrelevant background noise (Liawrungrueang et al., 2023). Secondly, the scarcity of medical datasets further exacerbates training challenges. Most existing studies rely on small-scale datasets, and the annotation process requires the expertise of professional radiologists, leading to high costs (Ke et al., 2025). This data limitation makes models prone to overfitting, and although the YOLO series models (such as YOLOv5 and YOLOv8) have incorporated improvements such as attention mechanisms (Duan et al., 2024) and residual modules (Wang et al., 2025), they still struggle to fully learn the spatial associations of complex pathological features under limited data conditions (He et al., 2023; Xu et al., 2025). Therefore, under the constraint of scarce medical datasets, developing novel YOLO architectures that can focus on critical texture regions while maintaining training efficiency and diagnostic accuracy has become a key breakthrough for advancing automated LDD diagnosis performance (Liawrungrueang et al., 2025).

To address the technical bottlenecks present in the field of LDD diagnosis, we propose an innovative solution—the YOLOv9 with Attention-Integrated Diagnostics (YOLOv9-AID) model. This model significantly enhances diagnostic performance through multi-dimensional technical innovations. First, drawing inspiration from the Extra DepthWise (ExtraDW) variant of the Universal Inverted Bottleneck (UIB) module, we optimize the RepNCSPELAN4 block in YOLOv9, improving model accuracy while increasing the overall network parameter size by only 0.4 MB. Second, in terms of network architecture, the Spatial-Channel Synergistic Attention (SCSA) mechanism is innovatively embedded at the 5th and 11th layers, thereby strengthening the model’s feature representation capacity. Third, at the loss function level, the SlideLoss strategy is introduced to effectively balance the contributions of high-quality and low-quality samples during model training. These designs significantly enhance the model’s ability to capture subtle pathological features of the intervertebral disc (Duan et al., 2024). Experimental results demonstrate that YOLOv9-AID comprehensively outperforms existing networks in key object detection metrics, achieving a mean average precision (mAP) of 82.8% at the IoU threshold 0.5 (mAP50), an overall precision of 80.3% (with 92.9% for Schmorl’s node detection), and a recall improvement of 5% in general (with a 15.1% increase specifically for Schmorl’s node detection). Notably, the model exhibits exceptional robustness in cross-dataset evaluations, which is attributed to the synergistic optimization of the attention mechanism and improved loss function. These technological advancements provide a novel and effective solution for the simultaneous and precise detection of Pfirrmann grading, disc herniation, HIZ, and Schmorl’s nodes. The subsequent sections will elaborate in detail on the model architecture design principles and the experimental validation process.

## 2 Materials

### 2.1 General guidelines

This study was conducted and reported in accordance with the Standards for Reporting of Diagnostic Accuracy (STARD) guidelines (see Supplementary Materials) and adhered to the principles of the Declaration of Helsinki. Ethical approval was obtained from the Institutional Review Board (EC-LCKY2025043). As a retrospective study involving no additional interventions or disclosure of personal data, the requirement for informed consent was waived.

### 2.2 Data collection and research design

This retrospective study included 222 patients (age ≥ 18 years) who were admitted between January 2023 and December 2024 with complaints of low back pain or radicular leg pain. A study has demonstrated the equivalence of sagittal T2-WI in the diagnosis of LDH (Alomari et al., 2014). For each patient, five standardized sagittal slices were systematically selected from their imaging data, resulting in a total of 1,110 image samples. All images were de-identified in compliance with medical data privacy regulations. Inclusion criteria were as follows: (1) clinically diagnosed with lumbar degenerative disease with radicular symptoms; (2) completion of a standardized lumbar MRI examination protocol. Exclusion criteria included (1) history of spinal trauma; (2) history of malignant tumors or spinal metastases; (3) previous lumbar spine surgery; (4) congenital scoliosis with Cobb angle greater than 10°; and (5) presence of imaging artifacts that hinder anatomical interpretation.

To rigorously evaluate the model’s performance and generalizability, an internal-external validation strategy was adopted. Among the selected patients, 202 cases (1,010 images) were randomly assigned to the internal dataset, which was split into training, validation, and test sets at a ratio of 8:1:1 for model development and internal validation. The remaining 20 cases (100 images) constituted the external dataset, which was exclusively used as the test set to evaluate the model’s generalization ability.

It is important to note that to simulate real-world clinical scenarios involving cross-vendor and multi-center data, both the internal and external datasets were acquired using MRI scanners from different manufacturers.

Internal Dataset: Images were acquired using the uMR 660 1.5T MRI scanner (United Imaging Healthcare, Shanghai) following the routine clinical lumbar imaging protocol. External Validation Dataset: Images were obtained using the Siemens Avanto 1.5T MRI scanner (Siemens Healthineers, Erlangen, Germany).

Both datasets were collected at the same institution (The First Affiliated Hospital of Jishou University, Jishou University, Jishou, China) to control for population differences while introducing technical heterogeneity through the use of different MRI systems. There was no overlap between the two patient groups. Table 1 summarizes the detailed MRI acquisition parameters for both datasets.

**TABLE 1:** MRI acquisition parameters for T2-weighted sagittal scans.

Classes	Internal dataset	External dataset
TR/TE	2335/94 m	3200/82 m
Sagittal matrix	456 × 561	384 × 384
Sagittal slice thickness	4 mm	4 mm
Sagittal spacing between slices	4.4 mm	4.4 mm

The standardized selection ensures a representative clinical cohort and provides high-quality, interpretable imaging data for subsequent analysis. The design of the internal-external validation strategy aims to assess the robustness of the developed model in the context of cross-device domain transfer and ensures its clinical applicability in heterogeneous real-world environments.

### 2.3 Dataset labeling and reference standard

Prior to annotation, we extracted five eligible DICOM sequences per patient and converted them into PNG format using Python scripts. All images underwent blinded evaluation and annotation by two board-certified radiologists ( > 6 years of experience) working independently. Discrepant assessments were arbitrated by a senior radiologist. All annotations were performed using the LabelImg software.

**FIGURE 1:**
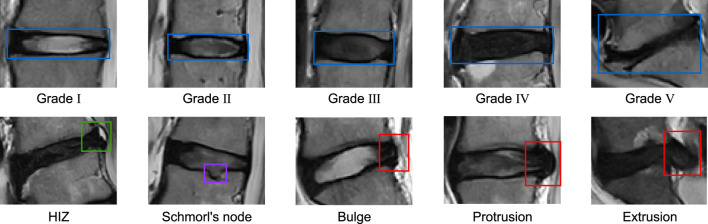
Examples of annotation types for lesions of interest in the dataset.

The diagnostic annotation reference standards used are as Figure 1: LDD was graded using the Pfirrmann classification system, which categorizes degeneration into five levels: Grade I (homogeneously hyperintense nucleus pulposus [NP] with clear NP-annulus demarcation and normal disc height), Grade II (mild NP hypointensity ± horizontal banding but preserved boundary definition), Grade III (marked NP hypointensity with blurred NP-annulus interface and potential mild height reduction), Grade IV (complete NP-annulus integration with signal extinction), and Grade V (diffuse hypointensity and severe disc collapse) (Pfirrmann et al., 2001). Disc herniation was classified according to the 2014 consensus nomenclature by the North American Spine Society (NASS), American Society of Spine Radiology (ASSR), and American Society of Neuroradiology (ASNR), defining bulge as annular extension ≤ 3 mm beyond vertebral margins, protrusion as focal extension > 3 mm with a base wider than the extruded portion, and extrusion as herniated material exceeding the base width in superior-inferior dimension. HIZ were identified as focal hyperintense regions within the hypointense annulus fibrosus on T2-WI, distinct from the NP and exhibiting higher signal intensity. At the same time, Schmorl’s nodes were characterized as intravertebral herniations manifesting as endplate depressions or intraosseous nodular lesions (Teraguchi et al., 2020).

Based on the above reference standards, we categorized all intervertebral discs in our study into ten distinct pathological classes. The distribution of these classes across the training set, validation set, internal test set, and external test set is detailed in Table 2.

**TABLE 2:** Composition of dataset.P1 to P5 represent the five grades (Grade I to Grade V) of the Pfirrmann classification system. HIZ refers to high-intensity zone. S1 denotes disc bulge, S2 indicates disc protrusion, S3 signifies disc extrusion, and Schmorl refers to Schmorl’s node.

Class of defects	Training set	Validation set	Internal test set	External test set	Total
P1	1495	184	221	264	2164
P2	2391	282	275	313	3261
P3	1318	156	155	192	1821
P4	1299	183	168	102	1752
P5	138	24	7	16	185
HIZ	254	22	23	15	314
S1	930	83	117	127	1257
S2	796	61	98	44	999
S3	458	21	53	29	561
Schmorl	180	145	16	22	363

## 3 Methods

**FIGURE 2:**
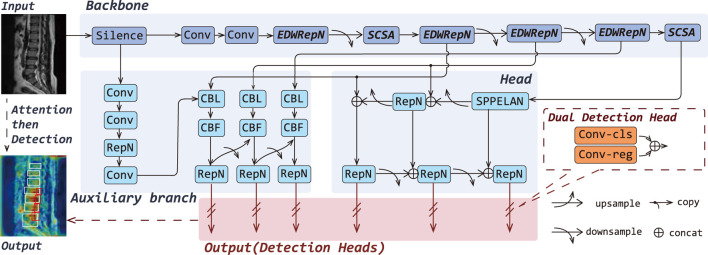
Overall architectures of the YOLOv9-AID. The italicized portions in Backbone indicate our proposed improvements.

To enhance the fine-grained lesion recognition capability of YOLOv9 in medical imaging, particularly for the challenging task of detecting LDD in MRI, which involves small targets, low contrast, and strong interference, we propose a novel architecture named YOLOv9-AID, as illustrated in Figure 2.

Specifically, we design the EDWRepNCSPELAN4 module to improve feature fusion within the backbone network, enabling more effective extraction and integration of multi-scale contextual information. We also introduce an SCSA module, which incorporates structure-aware enhancement into critical layers, thereby reinforcing the model’s focus on key spatial and semantic features. Furthermore, we incorporate a SlideLoss adaptive weighted loss function, which dynamically addresses the imbalance between easy and hard samples during training to stabilize convergence and improve detection accuracy. Detailed descriptions of each proposed component are provided in the following sections.

### 3.1 Improved feature extraction-fusion module

**FIGURE 3:**
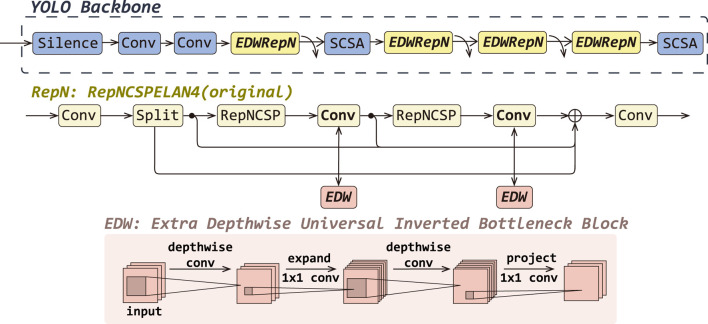
The detailed improvements of EDWRepNCSPELAN4 (EDWRepN) within the backbone network, including a structural comparison between the original RepNCSpELAN4(RepN) and EDWRepNCSPELAN4, as well as the architecture of the ExtraDW block.

In this work, we propose EDWRepNCSPELAN4 as a novel module to replace the original RepNCSPELAN4 in the backbone network, thereby constructing a feature extraction architecture that balances lightweight design with strong representational capacity. RepNCSPELAN4 combines the split–concatenate strategy of the cross-stage partial network (CSPNet) with the gradient-path optimized aggregation of the efficient layer aggregate network (ELAN) to balance learning capacity and multi-scale feature fusion. Specifically, as shown in RepN in Figure 3, given an input feature map $X\in R^{W\times H\times C}$ , RepNCSPELAN4 first applies a 1×1 convolution to produce $y_{0}$ . Next, two cascaded stages of RepNCSPELAN blocks—each consisting of a Replicated Neighboring Channel-Spatial Pooling unit followed by a 3×3 convolution—generate progressively deeper features $y_{1}$ and $y_{2}$ . Finally, $\left\{y_{0},y_{1},y_{2}\right\}$ are concatenated along the channel dimension and merged through a final 1×1 convolution, which produces the output of the module as shown in Formula 1, 2, 3, 4.

$$y_{0}=Conv_{1\times 1}\left(X\right),$$ (1)

$$y_{1}=Conv_{3\times 3}\left(RepNCSP\left(y_{0}\right)\right),$$ (2)

$$y_{2}=Conv_{3\times 3}\left(RepNCSP\left(y_{1}\right)\right),$$ (3)

$$Output=Conv_{1\times 1}\left(Concat\left(y_{0},y_{1},y_{2}\right)\right),$$ (4)

where $Conv_{1\times 1}\left(\cdot \right)$ and $Conv_{3\times 3}\left(\cdot \right)$ denote 1×1 and 3×3 convolutional operations, respectively; ResNCSP represents a Residual Neighboring Channel-Spatial Pooling block (a variant of residual structure for feature enhancement); and Concat refers to the channel-wise concatenation operation.

To enhance representational power without substantially increasing computation, we replace each 3×3 convolution in RepNCSPELAN4 with the ExtraDW block variant of the Universal Inverted Bottleneck (UIB) module, yielding EDWRepNCSPELAN4. The ExtraDW block follows a DepthWise–PointWise–DepthWise–PointWise sequence as illustrated in Figure 3: an initial depthwise 3×3 convolution expands the receptive field, a pointwise 1×1 convolution expands channel dimensionality by a factor α , a second depthwise 3×3 convolution further enriches spatial context, and a final pointwise 1×1 convolution projects features back to the original channel count. Formally, for an input $X_{1}\in R^{B\times W\times H\times C}$ , the UIBBlock computes as Fomulas 5, 6, 7, 8.

$$U_{1}=ReLU6\left(BN\left(DWConv_{3\times 3}\left(X_{1}\right)\right)\right),$$ (5)

$$U_{2}=ReLU6\left(BN\left(PWConv_{1\times 1}\left(U_{1}\right)\right)\right),$$ (6)

$$U_{3}=ReLU6\left(BN\left(DWConv_{3\times 3}\left(U_{2}\right)\right)\right),$$ (7)

$$U_{\mathrm{out}}=BN\left(PWConv_{1\times 1}\left(U_{3}\right)\right),$$ (8)

where ReLU6(⋅) is the activation function, BN(⋅) denote BatchNorm2d, a common batch normalization method, $DWConv_{3\times 3}\left(\cdot \right)$ and $PWConv_{1\times 1}\left(\cdot \right)$ represent depthwise convolution 3×3 and pointwise convolution 3×3 , respectively.

Finally, the output of the EDWRepNCSPELAN4 is shown in Formula 9.

$$Y=Conv_{1\times 1}\left(Concat\left(y_{0},U_{\mathrm{out}}^{\left(1\right)},U_{\mathrm{out}}^{\left(2\right)}\right)\right),$$ (9)

where $U_{\mathrm{out}}^{\left(1\right)}$ and $U_{\mathrm{out}}^{\left(2\right)}$ are the outputs of the first and second EDW blocks, respectively;

By embedding ExtraDW Block into the residual paths of the CSP-ELAN fusion framework, EDWRepNCSPELAN4 deepens the network and enlarges the effective receptive field, while keeping parameter and FLOP overhead modest. This hybrid design leverages the spatial-channel decoupling of depthwise separable convolutions and the bottleneck expansion principle to produce richer, more discriminative feature representations. Consequently, the model can focus more precisely on salient target regions, suppress background interference, and ultimately achieve higher detection accuracy without sacrificing real-time performance.

### 3.2 Spatial-channel attention boost

**FIGURE 4:**
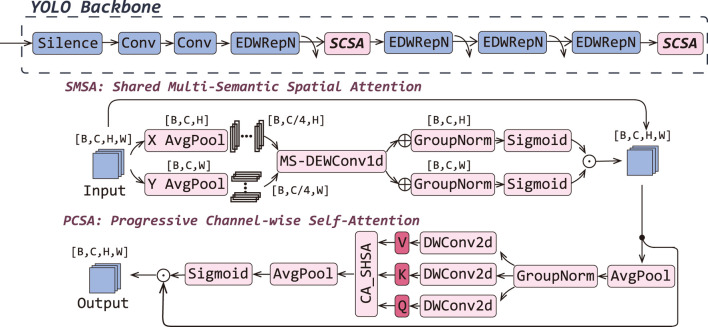
Overall architectures of the YOLOv9-AID. The italicized portions in Backbone indicate our proposed improvements.

In the MRI-based diagnosis of LDD, challenges such as Schmorl’s nodes and HIZ detection pose significant challenges due to their small target size and low contrast. Concurrently, Pfirrmann grading and herniation classification face inherent limitations, including high inter-class similarity, complex background interference, and noise artifacts, all of which adversely impact recognition accuracy. To address these issues, we introduced SCSA at the 5th and 11th layers of YOLOv9 as shown in Figure 4. This novel module employs multi-scale depth-wise shared convolutions to capture multi-semantic spatial information, strategically weights the central disc region through attention allocation, and effectively integrates global contextual dependencies. Furthermore, it incorporates an input-adaptive self-attention mechanism to refine target-relevant channel features, thereby mitigating semantic discrepancies in spatial configurations while maintaining anatomical structure awareness.

SCSA comprises two complementary submodules:

Shared Multi-Semantic Spatial Attention (SMSA): Firstly, the input feature map $X\in R^{B\times C\times H\times W}$ is pooled along height and width to yield unidirectional descriptors $X_{H}\in R^{B\times C\times W}$ and $X_{W}\in R^{B\times C\times H}$ . Then, each descriptor is split into four channel groups ${\left\{X_{H}^{i},\;X_{W}^{i}\right\}}_{i=1}^{4}$ and passed through shared depth-wise 1D convolutions of varying kernel sizes (3, 5, 7, 9) to capture multi-semantic spatial cues. Finally, convolved sub-features are concatenated, normalized via GroupNorm (4 groups), and activated by sigmoid to produce spatial attention maps ${Attn}_{H}$ and ${Attn}_{W}$ . The refined spatial output is given by Formula 10.

$$X_{s}=Attn_{H}⊙Attn_{W}⊙X.$$ (10)

Progressive Channel Self-Attention (PCSA): The SMSA output $X_{s}$ is down-sampled with 7 × seven pooling to $X_{p}$ , then linearly projected via three depth-wise 1 × one convolutions into query Q , key K , and value V . Subsequently, single-head self-attention is computed as Formula 11.

$$X_{\mathrm{attn}}=Softmax\left(\frac{QK^{T}}{\sqrt{C}}\right)V,$$ (11)

capturing global context. A final pooling to a scalar per channel followed by sigmoid gating re-weights the spatial output to yield as Formula 12.

$$X_{c}=X_{s}⊙\sigma \left(Pool\left(X_{\mathrm{attn}}\right)\right).$$ (12)

By decoupling spatial and channel dimensions, leveraging multi-scale depth-wise convolutions, and integrating self-attention, SCSA expands the effective receptive field, adaptively emphasizes the central disc region, and reconciles semantic discrepancies—thereby improving detection of subtle pathologies (e.g., Schmorl’s nodes), disambiguating classes with high similarity (e.g., Pfirrmann grades), and suppressing background noise. Importantly, SCSA achieves this with minimal added complexity, striking a balance between accuracy, inference speed, and model size.

### 3.3 Optimization of the loss function

The loss function is a mathematical tool that quantifies the errors in model predictions and occupies a central role in deep learning. During the model training process, the loss function calculates the difference, or loss value, between the predicted output of the model and the actual labels, which provides an intuitive reflection of the model’s performance.

YOLOv9 employs the Intersection over Union (IoU) to measure the disparity between the predicted bounding box and the ground truth box. The calculation of IoU is defined as Formula 13

$$IoU\left(P,G\right)=\frac{area\left(P\cap G\right)}{area\left(P\cup G\right)},$$ (13)

where P is the predicted bounding box and G is the ground truth box. However, in traditional loss functions, all samples are assigned the same loss weight, which may lead to the model placing excessive focus on easy samples during the training process while neglecting harder samples.

Therefore, we introduce a new loss function, SlideLoss (Yu et al., 2024). This is an adaptive weighted loss function that effectively alleviates the imbalance between easy and hard samples by assigning different weights to samples within the IoU range. The weight function of SlideLoss is defined as in Formula 14

$$f\left(x\right)=\left\{\begin{array}{cc} 1\; & \text{ if }x\leq \mu -0.1\\ e^{1-\mu }\; & \text{ if }\mu -0.1<x<\mu \\ e^{1-x}\; & \text{ if }x\geq \mu \end{array}\right.,$$ (14)

where μ is the average value of the IoU for all bounding boxes.The SlideLoss function differentiates between easy and hard samples based on the IoU value and uses its average value, μ , as a threshold. Samples with an IoU less than μ are classified as negative samples, while those with an IoU greater than μ are classified as positive samples.

Since most image processing tasks are imbalanced, with easy samples far outnumbering hard samples, the SlideLoss function effectively addresses the sample imbalance issue, encouraging the model to focus more on the hard samples during training. At the same time, the automatic computation of the threshold μ reduces the difficulty of manually setting hyperparameters and enhances the model’s adaptability. Thus, the inclusion of the SlideLoss function allows the model to better learn the features of hard samples, improving its generalization capability.

## 4 Experiments and results

### 4.1 Environment configuration and parameter settings

All experiments in this study were conducted on a Windows 10 operating system with an Intel i9-10920X CPU and NVIDIA GeForce RTX 3090 GPU. The software environment consisted of Python 3.8, PyTorch 2.3.1, and CUDA 11.8. To ensure comparability across different algorithms, we maintained identical training parameters throughout all evaluations. Input images were resized to 640 × 640 pixels with a batch size of 8. We employed Stochastic Gradient Descent (SGD) as the optimizer, configured with an initial learning rate of 1e-2, momentum of 0.937, and weight decay of 0.0005. Aligning with clinical requirements for high diagnostic precision, we set the validation IoU threshold to 0.6 and confidence threshold to 0.4 during inference. These standardized configurations were established to ensure reliable performance benchmarking while reflecting real-world medical imaging application scenarios.

### 4.2 Evaluation metrics

The performance of the YOLOv9-AID network is comprehensively evaluated from three critical dimensions: detection accuracy, computational efficiency, and architectural complexity. To quantify diagnostic precision and reliability, we employ established metrics including Precision, Recall, mAP, and F1-Score (F1). The algorithm’s real-time processing capability is assessed through Frames Per Second (FPS) measurements. Furthermore, model complexity is systematically characterized by analyzing the cardinality of trainable parameters, serving as an indicator of computational resource requirements and deployment feasibility. This tripartite evaluation framework ensures a rigorous assessment of the model’s practical applicability in time-sensitive detection scenarios.

The calculation formulas for P, R, mAP, F1 and FPS are as Formula 15, 16, 17, 18, 19.

$$Precision=\frac{TP}{TP+FP},$$ (15)

$$Recall=\frac{TP}{TP+FN},$$ (16)

$$mAP=\frac{1}{N}\overset{n}{\underset{i=1}{\sum }}\int_{0}^{1}P\left(R\right)dR,$$ (17)

$$F1=2\times \frac{P\times R}{P+R},$$ (18)

$$FPS=\frac{1}{processingtimeperframe},$$ (19)

where True Positives (TP) represent the count of positive-class samples correctly identified as positive. False Positives (FP) denotes the number of negative-class samples erroneously classified as positive, while False Negatives (FN) indicate the misclassification of positive-class samples as negative. The symbol N corresponds to the total number of categories in the classification system. P(R) represents the precision at specific recall levels, and d(R) denotes the differential of Recall, indicating the incremental change during the integration process.

### 4.3 Experimental results and analysis

#### 4.3.1 Overall performance evaluation

We selected various models with comparable parameter sizes (approximately 50 MB) and conducted training and testing based on our internal dataset. As shown in Table 3, across evaluation metrics including Precision, Recall, mAP50, mAP50–95, and F1-score, our model consistently demonstrated clear advantages over mainstream models. Specifically, in terms of Precision, our YOLOv9-AID model achieved 80.3%, surpassing RTDETR-x by 22%, which significantly enhances the efficiency of medical image processing. Moreover, our model attained a Recall of 82.1%, representing a 7.9% improvement compared to the baseline YOLOv9c. This high recall rate indicates that YOLOv9-AID possesses outstanding detection capabilities across almost all relevant categories, effectively minimizing the risk of missed detections. Consequently, in terms of F1-score—an indicator that comprehensively balances Precision and Recall—YOLOv9-AID achieved the highest score among all models evaluated, reaching 81%, which is 20% higher than that of the conventional RTDETR-x.

**TABLE 3:** The results of various different algorithms on internal datasets of this article. The best result in each type are in the bold font.

Method	Precision	Recall	mAP50	mAP50-95	F1-score
RTDETRx	0.583	0.634	0.606	0.39	0.61
YOLOv8x	0.751	0.742	0.755	0.526	0.74
YOLOv9c	0.778	0.771	0.792	0.541	0.77
YOLOv10x	0.727	0.713	0.729	0.501	0.71
YOLOv11x	0.75	0.737	0.749	0.513	0.74
YOLOv12x	0.775	0.793	0.787	0.533	0.78
Ours	**0.803**	**0.821**	**0.828**	**0.57**	**0.81**

Meanwhile, as shown in Table 4, our model also has a very good effect in the external test set. Among them, Recall, mAP50 and F1-score are all the highest in the compared models, which are 75%, 70.8% and 71% respectively. In Precision, it decreased by 2.6% compared to the best-performing YOLOv10x, but was still 2% higher than the YOLOv9c baseline model.

**TABLE 4:** The results of various different algorithms on external datasets of this article. The best result in each type are in the bold font.

Method	Precision	Recall	mAP50	mAP50-95	F1-score
RTDETRx	0.557	0.584	0.558	0.286	0.58
YOLOv8x	0.627	0.717	0.671	0.364	0.66
YOLOv9c	0.666	0.723	0.695	**0.388**	0.69
YOLOv10x	**0.712**	0.675	0.687	0.378	0.68
YOLOv11x	0.649	0.698	0.668	0.372	0.66
YOLOv12x	0.68	0.733	**0.708**	0.389	0.69
Ours	0.686	**0.75**	**0.708**	0.385	**0.71**

As demonstrated more clearly in Table 5, YOLOv9-AID consistently surpassed the baseline model YOLOv9c across all evaluation metrics. In terms of Precision, the majority of categories showed substantial improvements, with the Schmorl category exhibiting a particularly noteworthy increase from 72.5% to 92.9%, underscoring the effectiveness of our method in mitigating false positives. With regard to Recall, the proposed method also revealed marked advancements; for example, in the S2, S3, and Schmorl categories, Recall rates rose from 66.3%, 67.9%, and 66.1%–78.6%, 77.4%, and 81.2%, respectively, significantly reducing the risk of missed detections. Moreover, for the mAP50 metric, the overall score increased from 79.2% to 82.8%, with YOLOv9-AID either outperforming or matching YOLOv9c across most categories. Under the more stringent mAP50-95 metric, the average enhancement reached 2.9%, further confirming the robustness and precision of the proposed method across varying IoU thresholds. Concerning the F1-score, a pivotal evaluation metric, YOLOv9-AID improved from 77% to 81%, reflecting a more optimal equilibrium between Precision and Recall. Notably, in small-target and complex-background categories, such as S1, S2, and S3, the proposed method demonstrated superior stability in detection performance, highlighting its strong adaptability to diverse scenarios. Overall, the experimental results convincingly establish that the proposed method offers exceptional generalizability and substantial performance gains in multi-category detection tasks. To further elucidate the specific contributions of each module to the overall performance, comprehensive ablation studies will be presented in the following section.

**TABLE 5:** A detailed comparison between YOLOv9c and our model.The best results in each type are in the bold font.

	Precision		Recall		mAP50		mAP50-95	
Class	YOLOv9	Ours	YOLOv9	Ours	YOLOv9	Ours	YOLOv9	Ours
P1	0.887	**0.933**	**0.946**	0.941	0.946	**0.95**	0.691	**0.712**
P2	0.908	**0.913**	0.902	**0.92**	0.91	**0.923**	0.671	**0.68**
P3	**0.817**	0.806	0.92	0.91	**0.882**	0.88	**0.66**	0.651
P4	0.905	**0.928**	0.911	**0.917**	**0.939**	0.932	**0.725**	0.713
P5	**0.75**	**0.75**	**0.857**	**0.857**	**0.87**	0.852	0.592	**0.642**
HIZ	**0.812**	0.7	0.565	**0.609**	**0.69**	0.653	0.423	**0.441**
S1	**0.66**	0.65	0.607	**0.684**	0.594	**0.688**	0.37	**0.412**
S2	0.67	**0.694**	0.663	**0.786**	0.68	**0.757**	0.439	**0.474**
S3	0.648	**0.732**	0.679	**0.774**	0.661	**0.773**	0.402	**0.486**
Schmorl	0.725	**0.929**	0.661	**0.812**	0.748	**0.873**	0.433	**0.492**
All	0.778	**0.803**	0.771	**0.821**	0.792	**0.828**	0.541	**0.57**

**FIGURE 5:**
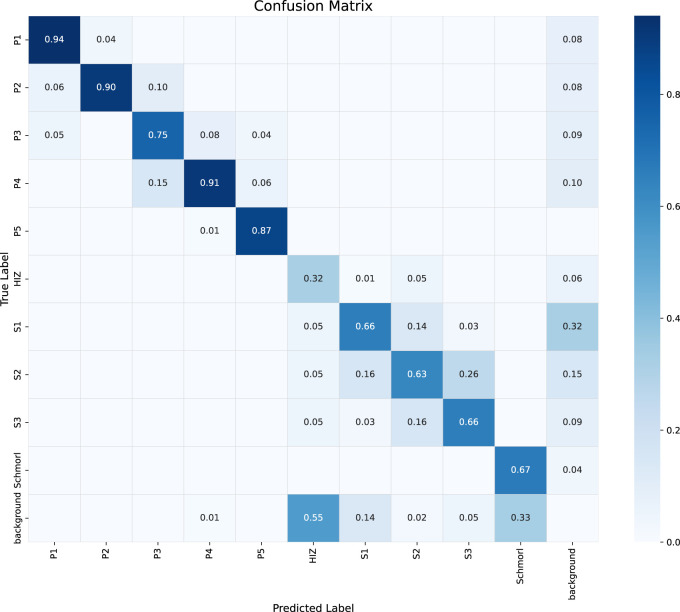
Confusion matrix of the classification performance for the proposed YOLOv9-AID model on the test set. The diagonal elements represent the recall for each class, while the off-diagonal elements indicate misclassifications between classes.

To analyze the model’s classification performance in more detail, we present the confusion matrix on the test set in Figure 5. As shown in the matrix, the model performs excellently on categories P1, P2, P4, and P5, with recognition accuracies (the values on the diagonal) all exceeding 0.87. However, the model exhibits noticeable confusion when distinguishing between the staging-related classes S1, S2, and S3. For instance, 26% of S2 cases were incorrectly classified as S3. Furthermore, the HIZ class has a relatively low recognition accuracy of 0.32 and is prone to misclassification. This confusion is likely attributable to the high similarity in visual features and the subtle grading boundaries among these lesions on MRI images, which poses a significant challenge for precise classification.

#### 4.3.2 Ablation experiment

In this section, we designed a series of ablation experiments based on our dataset to validate the effectiveness and necessity of each individual module proposed in the YOLOv9-AID framework. Specifically, we examined the contributions of the EDWRepNESPELAN4 structural enhancement, the SCSA (Spatial–Channel Synergistic Attention) mechanism, and the SlideLoss function. Each of these modules was integrated separately and in combination into the baseline network YOLOv9c, and all models were trained under identical settings while maintaining similar parameter sizes. The detailed experimental results are shown in Table 6, which reveals the distinct impacts of these three modules on Precision, Recall, mAP50, FPS, and overall model complexity. The findings indicate that while each component contributes differently, all three collectively enhance the diagnostic performance, demonstrating their indispensable roles in optimizing both detection accuracy and computational efficiency.

**TABLE 6:** Quantitative results of different methods on internal dataset. The best result in each type are in the bold font.

EDWRepNESPELAN4	SCSA	Slide loss	Precision	Recall	mAP50	FPS	Parameters
			0.778	0.771	0.792	40	**50.72M**
✓			0.791	0.782	0.803	40	51.14M
	✓		0.78	0.786	0.804	**42**	50.73M
		✓	0.764	0.785	0.782	38	**50.72M**
✓		✓	0.787	0.82	0.817	39	51.14M
	✓	✓	0.787	0.737	0.77	42	50.73M
✓	✓		0.765	0.769	0.793	37	51.15M
✓	✓	✓	**0.803**	**0.821**	**0.828**	37	51.15M

For the mAP50 metric, the contributions of SlideLoss, EDWRepNESPELAN4, and SCSA increase sequentially, and their combination leads to a total improvement of 3.6%, indicating that the integration of all three modules effectively enhances detection accuracy. Notably, the attention mechanism introduced by SCSA significantly improves FPS, and despite the added architectural complexity, the overall drop in FPS is limited to just 3, demonstrating that the coordinated integration of the three modules does not compromise inference speed. Both EDWRepNESPELAN4 and SCSA attention modules contribute meaningfully to improvements in precision and recall, while the dynamic weight adjustment introduced by SlideLoss amplifies the strengths of the attention mechanisms without increasing model complexity. Compared to the version with only EDWRepNESPELAN4 and SCSA, the inclusion of SlideLoss results in a 3.8% increase in precision and a 5.2% increase in recall.

#### 4.3.3 Visualization of the contribution of the attention mechanism

To more intuitively illustrate the contribution of the attention mechanism module to image recognition and processing, we utilized Gradient-weighted Class Activation Mapping (Grad-CAM) for heatmap visualization. For each image, four versions were presented: the original image, the image with ground-truth bounding boxes, the YOLOv9c prediction with its corresponding Grad-CAM heatmap, and the YOLOv9-AID prediction with its Grad-CAM heatmap. Two samples from the internal dataset and two from the external dataset are used for demonstration. In the Grad-CAM heatmaps, red regions indicate areas of high model attention—regions the algorithm deems important for decision-making. The closer these red regions are to actual pathological areas, the more efficient the image processing is, leading to more accurate detection outcomes.

**FIGURE 6:**
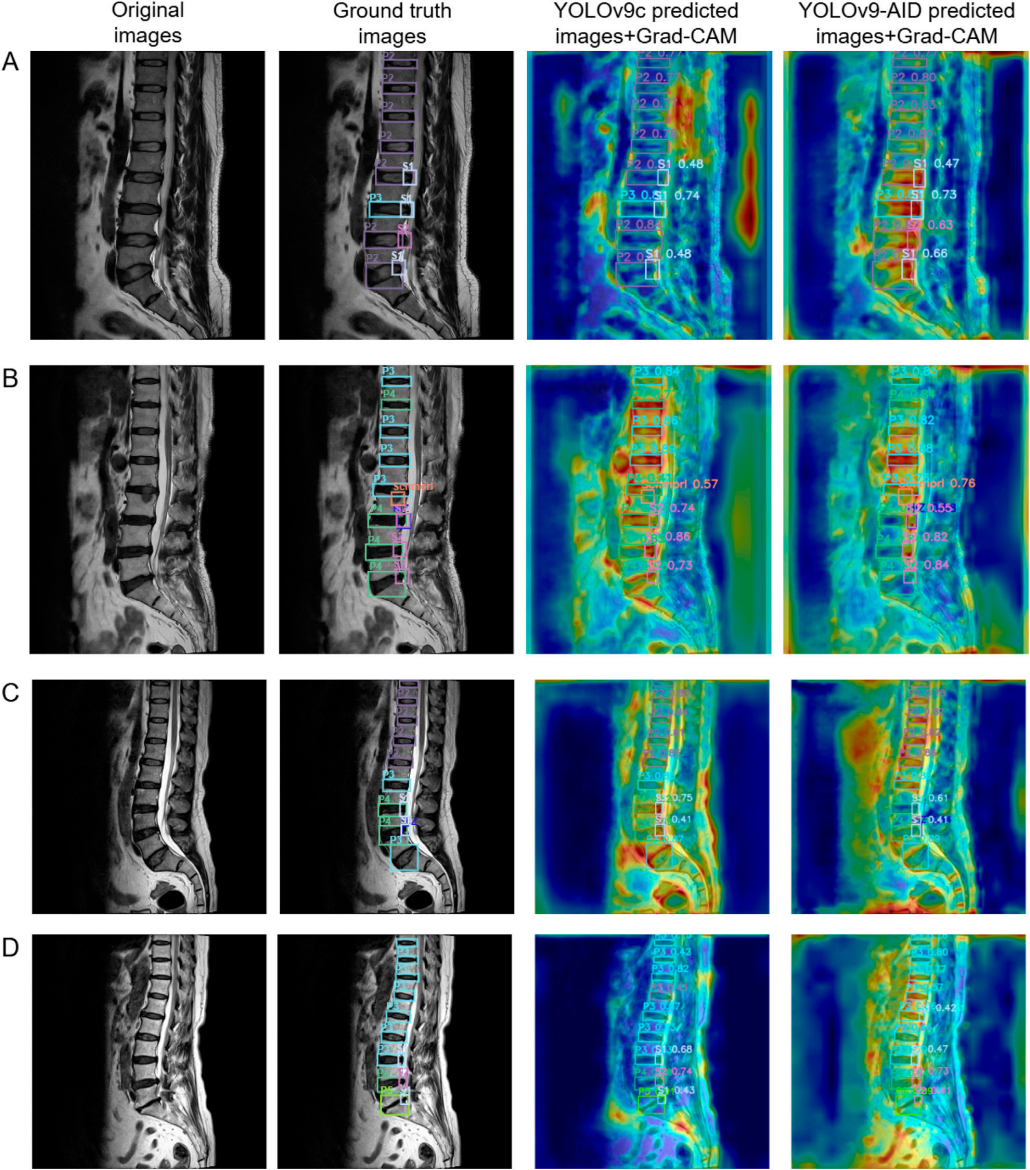
Comparative Panel of Internal and External Test Set Examples. The first column on the left displays the original images. The second column presents the original images with ground truth bounding boxes. The third column shows heatmap images of predicted bounding boxes from YOLOv9c. The fourth column illustrates heatmap images of predicted bounding boxes from YOLOv9-AID. Samples A and B are derived from the internal dataset, while samples C and D originate from the external dataset. Grad-CAM: Gradient-weighted Class Activation Mapping.

As shown in Figure 6, we selected four representative cases (A–D) for visualization. With the integration of YOLOv9-AID, the algorithm demonstrated enhanced focus on lesion regions, characterized by clearer boundaries and significantly reduced interference from irrelevant areas. These improvements suggest that our model exhibits substantially increased capability in identifying critical structures within medical images, thereby enhancing both accuracy and reliability. The comparison between ground-truth and predicted bounding boxes further supports these findings. For instance, in internal dataset image A, YOLOv9-AID increased the confidence score for the lower S1 subtype from 48% to 66%. In image B, also from the internal dataset, YOLOv9-AID successfully detected a High-Intensity Zone (HIZ) that YOLOv9c failed to identify. Our model also performed well on external datasets with greater image variability. In external image C, YOLOv9-AID detected an HIZ subtype missed by YOLOv9c. In external image D, it raised the confidence score of the upper P3 subtype from 42% to 80%. These experimental results strongly demonstrate the superior precision and efficiency achieved by our proposed model.

## 5 Discussion

Our research has demonstrated the feasibility of introducing an attention mechanism into YOLOv9c for medical image detection. Although there is a newer version, YOLOv12x, after testing on our internal dataset, it was found that its performance in the medical field was not satisfactory. YOLOv9c had the best initial performance, so we decided to use it as the basis for optimization. After a horizontal comparison of RTDETRx, YOLOv8x, YOLOv9c, YOLOv10x, YOLOv11x, and YOLOv12x, the YOLOv9-AID model proposed by us was found to be the most suitable.

In clinical diagnosis and treatment, the MRI assessment system for LDD emphasizes multi-dimensional value: the Pfirrmann grading (1-5 levels) serves as the gold standard. By evaluating the signal intensity of the nucleus pulposus, the height of the intervertebral disc, and structural characteristics, it can effectively distinguish early degeneration (II-III levels) from advanced degeneration (IV-V levels), providing a key basis for predicting disease progression and making surgical decisions (Soydan et al., 2024); higher grades (IV-V levels) are highly correlated with the loss of intervertebral disc height and endplate damage (Cheung et al., 2023). In terms of morphological changes, protrusion/extension/extrusion is gradientally associated with the risk of nerve root compression. Among them, protrusion and extrusion directly compress the nerve structure and are closely related to sciatica, motor dysfunction, and surgical indications (Patel et al., 2024). The high signal area (HIZ), as an imaging marker of annulus fibrosus tear, is significantly associated with lumbar pain (OR = 28.15), suggesting the risk of inflammatory factor release and nerve fiber infiltration (Yang et al., 2023); Schmorl nodules represent a special type of protrusion of the nucleus pulposus through the endplate into the vertebral body. Although they are mostly asymptomatic, acute bone marrow edema during the acute phase can cause mechanical lumbar pain (Lawan et al., 2021). Algorithms developed for MRI images are particularly important because MRI significantly outperforms X-rays in detecting Schmorl nodules, especially in clearly showing the edema signal on T2-weighted images (Cao et al., 2020).

In current clinical practice, there are observer-to-observer differences in Pfirrmann grading (Kappa = 0.66–0.89), the boundaries of the protrusions are unclear, and there is no unified standard for the various forms of HIZ and Schmorl nodules. Moreover, traditional manual interpretation takes 14–19 min per case (Compte et al., 2023b). YOLOv9-AID integrates degeneration grading, protrusion type, HIZ and Schmorl nodules’ multi-dimensional indicators into one, significantly shortens the interpretation time, and improves consistency through a standardized process, providing a set of efficient and reliable automated assessment solutions for lumbar degeneration in clinical practice.

In this study, the model demonstrated high classification accuracy across multiple categories (e.g., over 0.87 for P1, P2, P4, and P5), indicating its capability to identify key imaging features and enhance diagnostic reliability effectively. This high accuracy not only reduces the workload and potential for human error for physicians analyzing large volumes of images, particularly in highly repetitive and large-scale scenarios, but also facilitates faster screening of complex lesions such as HIZ and Schmorl’s nodes, thereby advancing precision medicine. Furthermore, it provides technical support for automated triage and intelligent computer-aided diagnosis systems.

In this study, for the LDH detection task on both internal and external datasets, the YOLOv9c-AID model we proposed outperformed multiple mainstream single-stage detectors including RTDETR-x, YOLOv8x, and YOLOv12x in several key metrics. Firstly, on the internal dataset, YOLOv9-AID achieved outstanding results of 80.3%, 82.1%, 82.8%, 57.0%, and 81.0% in Precision, Recall, mAP50, mAP50-95, and F1-score respectively (Table 3). Compared with the baseline model YOLOv9c, Precision increased by 2.5%, Recall by 5%, and mAP50 by 3.6%, indicating that our model has significant advantages in medical image processing efficiency and false negative risk control. Similarly, on the external dataset, YOLOv9-AID maintained strong generalization ability, achieving a 68.6% precision, a 75.0% recall, and a 71.0% F1-score (Table 4), fully demonstrating the robustness of the model on heterogeneous data.

From the perspective of performance at the category level, YOLOv9-AID has achieved varying degrees of performance improvements in the detection of small targets (such as Schmorl nodules), complex backgrounds (such as HIZ), and common intervertebral disc regions (P1-P5, S1-S3) (Table 5). Particularly in the Schmorl category, Precision has increased from 72.5% to 92.9%, Recall has risen from 66.1% to 81.2%, and mAP50 has improved from 74.8% to 87.3%. This significant improvement indicates that our SCSA mechanism can effectively suppress false positives, while the improvement in Recall for subtypes such as S2 and S3 reflects the prominent role of SlideLoss’s dynamic weight adjustment in reducing missed detections. The average improvement of 2.9% under strict IoU thresholds (mAP50-95) further validates the model’s robustness in detection under different overlap standards.

Compared with recent algorithms published over the past 3 years in Table 7, our model outperforms the (Guinebert et al., 2022) benchmark in overall precision and recall, although it trails three 2024–2025 studies (Natalia et al., 2024; Zhang et al., 2024; Wang et al., 2025)that leveraged larger datasets (48,345, 3225, and 5664 images) and focused on Pfirrmann grading. Notably, in Pfirrmann subtypes, our precision for grades I and IV leads all comparators by only using 1100 images, demonstrating that multi-task detection (including HIZ, disc herniation, and Schmorl’s nodes), which is out of consideration in previous studies, can be achieved without compromising grading accuracy. Given the impracticality of developing separate algorithms for each lesion type in clinical practice, our YOLOv9-AID network offers a competitive, comprehensive solution to assist MRI-based diagnosis of LDD.

**TABLE 7:** Performance comparison of the proposed model with previous studies.

References	Method	Precision	Recall	The precision of Pfirrmann grade					Total images	No. of patients
				I	II	III	IV	V		
Guinebert et al. (2022)	YOLOv5x	75.0%	76.5%	-	-	-	-	-	3660	244
Natalia et al. (2024)	ResNet-50 + DeepLabv3	88.1%	-	68.3%	92.6%	79.0%	82.7%	76.2%	48,345	515
Zhang et al. (2024)	Faster R-CNN	-	-	68.5%	84.0%	85.1%	84.4%	81.8%	3225	217
Wang et al. (2025)	YOLOv5	88.0%	88.0%	92.0%	84.0%	90.0%	91.0%	78.0%	5664	472
Ours	YOLOv9-AID	80.3%	82.1%	93.3%	91.3%	80.6%	92.8%	75%	1100	220

Furthermore, through ablation experiments (Table 6), we can observe that the three modules EDWRepNESPELAN4, SCSA, and SlideLoss, each make different contributions to the detection performance and inference efficienSlideLossucing EDWRepNESPELAN4 alone can increase mAP50% to 80.3%, while merely adding SCSA can increase FPS to 42. When the three modules work together, mAP50 reaches 82.8%, Precision and Recall increase to 80.3% and 82.1% respectively, and the frame rate drops to only 37, fully demonstrating the complementarity of the three modules and the overall optimization effect. The Grad-CAM visualization (Figure 6) further visually shows that YOLOv9-AID pays higher attention to the lesion area and has clearer boundaries, indicating that the attention mechanism plays a key role in suppressing irrelevant background interference and improving the model’s reliability.

The first limitation of this study is that both the internal and external test sets were derived from a single medical center. Although different imaging devices with varying brands and scanning parameters were employed, patients from the same institution may share similar regional, ethnic, or socioeconomic backgrounds. This homogeneity could lead to a limited diversity in disease distribution, especially for rare or atypical cases such as Grade V degeneration, HIZ, Schmorl’s nodes, and disc extrusions, which were underrepresented in our dataset (Futoma et al., 2020). As a result, the model may have limited generalizability and reduced detection performance for such uncommon cases. To address this, our roadmap for future multicenter validation includes several key steps. First, we will design a standardized data collection protocol and secure Institutional Review Board (IRB) approval from each participating institution. Second, we will prospectively collect a large-scale dataset from multiple centers, ensuring diversity in MRI vendors (e.g., Siemens, GE, Philips), field strengths (1.5T, 3.0T), and patient demographics. Third, the evaluation of YOLOv9-AID will extend beyond diagnostic accuracy to include its impact on clinical workflow, such as measuring the reduction in radiologists’ reading time and its effect on diagnostic confidence. This comprehensive validation is crucial for assessing the model’s real-world robustness and clinical utility, paving the way for its responsible implementation in diverse healthcare environments (Singh et al., 2022). This approach is expected to enhance the model’s robustness and generalizability in cross-institutional settings, making it more applicable for real-world clinical use.

The second limitation lies in the exclusive use of sagittal T2-weighted MRI images for model training. While these images yielded satisfactory performance, they may not fully capture the three-dimensional morphology of HIZ lesions, such as linear or annular patterns of annular tears. Furthermore, relying solely on sagittal views may result in the omission of key axial features indicative of nerve root compression, including lateral recess stenosis or root effacement (Harada et al., 2020). Prior research has demonstrated that HIZ is not only a marker of LDD but also a critical imaging sign of discogenic low back pain, with signal characteristics often correlating with chemical radiculitis and foraminal stenosis (Yang et al., 2023). In future work, we aim to incorporate axial T2-weighted images to create a more comprehensive dataset. This will allow for more precise localization of annular fissures and better quantification of nerve compression, ultimately improving the clinical relevance and diagnostic accuracy of the model.

The third limitation is the lack of integration of clinical indicators into the diagnostic process. Existing evidence suggests that unimodal imaging models may fall short when evaluating complex or ambiguous cases. Clinical data—such as patient history, physical examination findings, and symptom descriptions—play a crucial role in disease assessment and personalized treatment planning. In future studies, we plan to develop a multimodal diagnostic framework that integrates imaging models with natural language processing systems (Zhou et al., 2023; Wu et al., 2023). For instance, while our YOLOv9-AID model might identify an HIZ on an MRI scan, an NLP module could simultaneously process the patient’s clinical notes to extract key phrases such as “sharp, localized back pain” or “symptoms worsened by flexion.” Correlating the imaging biomarker (HIZ) with the patient’s specific symptoms (textual data) would significantly enhance diagnostic confidence and provide a more holistic assessment. By combining radiological images with structured and unstructured clinical information, we aim to improve diagnostic performance and facilitate the semi-automated generation of structured radiological reports (Chiumento and Liu, 2024). This integration may help bridge the current “modality island” in medical AI research and support the translation of AI-assisted diagnosis from research to clinical practice.

## 6 Conclusion

This study has achieved remarkable results in the intelligent diagnosis of LDD using the YOLOv9-AID model: The ExtraDW optimized deep separable convolution structure reduces the number of parameters while improving the detection accuracy; the SCSA module significantly enhances the representation ability of small lesion features; the sliding loss strategy effectively balances the training weights of different quality samples. The experimental results show that YOLOv9-AID outperforms traditional YOLOv9 and similar methods in key indicators such as mAP50, detection accuracy, and recall rate, and demonstrates excellent generalization ability in external datasets. This model can provide rapid and accurate synchronous detection and grading diagnosis assistance for clinical practice. In the future, it can be further expanded to multi-modal image fusion and real-time diagnosis systems, providing more reliable technical support for the early screening and precise treatment of lumbar degeneration.

## Data Availability

The raw data supporting the conclusions of this article will be made available by the authors, without undue reservation.
